# ASGR1: an emerging therapeutic target in hypercholesterolemia

**DOI:** 10.1038/s41392-023-01319-5

**Published:** 2023-01-23

**Authors:** Wenqi Zhao, Suowen Xu, Jianping Weng

**Affiliations:** Department of Endocrinology, Institute of Endocrine and Metabolic Diseases, The First Affiliated Hospital of USTC, Division of Life Sciences and Medicine, Clinical Research Hospital of Chinese Academy of Sciences (Hefei), University of Science and Technology of China, Hefei, 230027 China

**Keywords:** Endocrine system and metabolic diseases, Therapeutics, Cardiology, Endocrine system and metabolic diseases

In a recent study published in *Nature*, Wang et al.^1^ discovered that inhibition of asialoglycoprotein receptor 1 (ASGR1) increased cholesterol efflux and thus lowered blood cholesterol and reduced atherosclerosis. This study offers an emerging new therapeutic target in hypercholesterolemia and its comorbidities and complications (such as fatty liver and atherosclerosis), which are major threats to public health.

Hypercholesterolemia represents an important risk factor for cardiovascular and metabolic diseases (CVMD). Cholesterol-lowering is the mainstream therapy for CVMD and several cholesterol-lowering targets have translated to clinical arena. For example, β-hydroxy β-methylglutaryl-CoA (HMG-CoA) reductase (HMGCR) mediates cholesterol synthesis in the liver and serves as the primary target of statins. In addition, acetyl-CoA, the building block for cholesterol synthesis, is partially produced by ATP-citrate lyase (ACLY) from citrate. ACLY inhibitors thus represent another class of cholesterol-lowering drugs. For example, Bempedoic acid is a prodrug which is converted into an active metabolite in the liver, then inhibits the activity of ACLY specifically in the liver (without muscle toxicity), thereby reducing acetyl-CoA-dependent cholesterol synthesis and clinically it has a good safety profile. The third category of cholesterol-lowering drugs are inhibitors of Niemann–Pick C1-like 1 (NPC1L1), such as Ezetimibe, which reduces the absorption of cholesterol by enterocytes. Physiologically, low-density lipoproteins (LDL) are recognized and bound by the LDL receptor (LDLR) and transported as LDL-cholesterol (LDL-C) into hepatocytes to initiate cholesterol clearance. Thus, LDLR is an appealing target for LDL-C lowering. Proprotein convertase subtilisin–kexin type 9 (PCSK9) binds to and induces the degradation of LDLR. Therefore, different mechanisms of PCSK9 inhibition, such as siRNAs, monoclonal antibodies, and anti-sense oligonucleotides (ASOs), inhibit LDLR degradation and increase LDLR-dependent cholesterol uptake by hepatocytes, thereby reducing circulating cholesterol level. Statins, with good efficacy and few adverse effects, are the first-line cholesterol-lowering drugs. However, some patients may experience statin intolerance and increased risk of myopathy and hyperglycemia, even overt diabetes mellitus. Also, residual inflammatory risk remains in statin-treated patients. Therefore, these circumstances necessitate the discovery of novel therapeutic targets and therapeutic agents to address the challenges.

Increasing cholesterol excretion by the biliary system maybe a tentative strategy. Cholesterol secretion into the biliary system is mediated by ATP binding cassette subfamily G member 5 (ABCG5) and ABCG8, which are regulated by liver X receptor (LXR). An additional advantage of upregulating LXR is that ATP binding cassette subfamily A member 1 (ABCA1) would be increased, which transfers cholesterol to high-density lipoprotein (HDL) to mount reverse cholesterol transport. However, only upregulating the function of LXR to increase cholesterol excretion and reverse cholesterol transport is not clinically useful, because sterol regulatory element binding protein 1 (SREBP1) upregulation resulting from LXR activation increases lipid synthesis. In a recent study, Wang et al.^1^ discovered that inhibition of asialoglycoprotein receptor 1 (ASGR1) (a GWAS identified gene associated coronary artery disease) could upregulate ABCG5, ABCG8, and ABCA1 by increasing LXRα, but inhibiting SREBP1, which is distinguished from canonical LXRα activation by pharmacological activators.

In 1971, Ashwell et al. discovered ASGR1 when studying the uptake of glycoproteins by hepatocytes.^2^ ASGR1 is a subunit of the asialoglycoprotein receptor which is mainly located on the surface of hepatocytes. It binds glycoproteins with galactose (Gal) or N-acetylgalactosamine (GalNAc) residues (with higher affinity for GalNAc) and then mediates the endocytosis and lysosomal degradation of these glycoproteins.^2^ Ashwell et al. estimated that there were 200,000 to 500,000 asialoglycoprotein receptors on the surface of each rat hepatocyte, and each receptor returns to the plasma membrane 5-7 min after its endocytosis.^2^ In summary, unique features of ASGR1, such as liver specificity, liver enrichment, and presence on hepatocyte surface render ASGR1 amenable to therapeutic exploitation. Pharmaceutically, emerging studies have coupled GalNAc to small-molecule drugs or ASOs to achieve targeted therapy in the liver. In addition, ASGR1 also served as the receptor or co-receptor for virus invasion of liver, such as SARS-CoV-2, which results in liver damage frequently observed in COVID-19 patients.

In 2016, a landmark study by Nioi et al.^3^ found that a loss of function variant of *ASGR1* caused by a noncoding 12-base-pair (bp) deletion (del12) reduced non-HDL cholesterol and protected against the development of atherosclerosis. In the same study, the authors also found that p. W158X, another loss of function variant of ASGR1, was associated with reduced levels of non-HDL cholesterol. This work resulted in an increased interest in the mechanism through which ASGR1 lowered blood cholesterol.

Earlier studies have suggested that ASGR1 reduction increases LDLR and thus lowers blood cholesterol. For example, Xie et al.^4^ generated *Asgr1*-deficient pigs and found that when fed a high-fat and high-cholesterol diet for 6 months, these knockout pigs had lower levels of non-HDL than control pigs. To determine the mechanism of action, Xie et al. analyzed the hepatic transcriptomic profile and in vivo cholesterol metabolism and found that ASGR1 deficiency downregulated HMGCR but upregulated hepatic LDLR. However, the study by Wang et al.^1^ found no difference in hepatic LDLR expression among *Asgr1*^+/+^, *Asgr1*^+/−^, and *Asgr1*^−/−^ mice. This result may be due to the different animal models, having different metabolic characteristics which were used in these studies. There is a relevant example that the LXR-IDOL-LDLR (IDOL, inducible degrader of low-density lipoprotein receptor) axis is both tissue- and species-specific and is not present in mouse liver. Wang et al.^1^ found that ASGR1 regulates LXR, and the effect of LXR on LDLR may differ between pig and mouse livers depending on the presence or absence of the LXR-IDOL-LDLR axis.

Some studies have suggested that the effect of ASGR1 on blood cholesterol is related to the role of SREBP in the nucleus (nSREBP1). Xu et al.^5^ found reduced nSREBP1 in *ASGR1*-knockout HepG2 cells and *Asgr1*-knockout mouse liver tissues. Similar findings were obtained in the study by Wang et al.^1^. However, both studies offer two complementary mechanisms by which ASGR1 affects nSREBP1. Specifically, Xu et al. showed that the downregulation of ASGR1 increased the expression of the insulin-induced gene 1 (INSIG1), which anchors SREBP1 to the endoplasmic reticulum and leads to a decrease in nSREBP1. In contrast, Wang et al.^1^ suggested that the reduction in ASGR1 resulted in the downregulation of mammalian target of rapamycin complex 1 (mTORC1) and the upregulation of AMP-activated protein kinase (AMPK), which inhibited SREBP1 expression and activation. Taken together, ASGR1 could possibly affect nSREBP1 through two different pathways as described and thus thereby regulating cholesterol synthesis. In addition, AMPK can also regulate the degradation of INSIG dependent on phosphorylation. Clearly, both mechanisms of actions of ASGR1 in regulating nSREBP1 activation are complementary.

In the study by Wang et al.^1^, the results showed that ASGR1 binds and delivers asialoglycoproteins to lysosomes via clathrin-mediated endocytosis; subsequently, amino acids released from glycoprotein breakdown by lysosomes activate lysosomal mTORC1 and inhibit activation of the fuel sensor AMPK. This leads to the activation of SREBP1, which promotes cholesterol synthesis. Activation of lysosomal mTORC1 and inhibition of AMPK also resulted in increased expression of BRCA1 and BARD1, which form a ubiquitin ligase (E3) complex. The increased expression of BRCA1 and BARD1 promotes degradation of LXRα and reduction of LXRα inhibits the expression of downstream target genes *ABCG5* and *ABCG8* and thus reduces cholesterol excretion into bile acids. It also inhibits *ABCA1* expression and thus reduces cholesterol trafficking to HDL. In conclusion, ASGR1 binds to desaloglycans and initiates a chain reaction that ultimately leads to an increase in intracellular cholesterol. When *ASGR1* acquired loss-of-function mutations or inhibited expression, the internalization of asialoglycoproteins is suppressed, leading to mTORC1 inhibition and AMPK activation. Further, AMPK destabilizes BRCA1 and BARD1 mediated LXRα degradation, leading to an increase in LXRα, which in turn upregulates the expression of *ABCA1* and *ABCG5/G8* by promoting reverse cholesterol transport and pumping cholesterol into the bile for fecal excretion, respectively. Furthermore, activated AMPK inhibits cholesterol synthesis by inhibiting nSREBP1. In summary, genetic or pharmacological inhibition of ASGR1 exerts dual biological functions to halt hypercholesterolemia by inhibiting cholesterol synthesis while promoting cholesterol efflux and excretion from the body. The study by Wang et al.^1^ also suggests that ASGR1 ligands, AAV-shRNAs (AAV, adeno-associated virus) and a monoclonal antibody can alter ASGR1-mediated regulation of cholesterol metabolism, thus providing efficient tools to manipulate the functionality of ASGR1 in the future.

ASGR1 has great potential for clinical application as a target for lowering blood cholesterol level. The notable advantage of targeting ASGR1 is that its functionality is amenable to therapeutic intervention. ASGR1 is a receptor localized on the surface of hepatocytes, which renders it possible to perform structure-based drug discovery to identify ligand analogs or small-molecule compounds that can competitively bind to and inactivate ASGR1 to increase cholesterol efflux. To achieve this goal, high-throughput or high-content drug screening based on the ASGR1 crystal structure is a feasible approach. Another advantage of cholesterol-lowering effects delivered by ASGR1 inhibition is that this approach has the potential to work in synergy with other cholesterol-lowering pathways. For example, dual inhibition of NPC1L1 and ASGR1 represents a promising combination therapy. Mechanistically, inhibition of NPC1L1 (by ezetimibe for example) inhibits cholesterol reabsorption. While, inhibition of ASGR1 can promote the excretion of cholesterol into bile acids. This synergistic mechanism may pave the way for future polypill formulations of ASGR1 inhibitors and NPC1L1 inhibitors and beyond. Additionally, ASGR1 is mainly expressed in the liver, which reduces the theoretical possibility of multiple organ adverse reactions caused by ASGR1 inhibition. Furthermore, since ASGR1 is a receptor for SARS-CoV-2 invasion of liver, inhibition of ASGR1 may reduce the risk of liver injury in COVID-19 patients. Moreover, ASGR1 inhibitors may also have potential health benefits beyond lowering blood cholesterol and increasing cholesterol efflux.

**Fig. 1:**
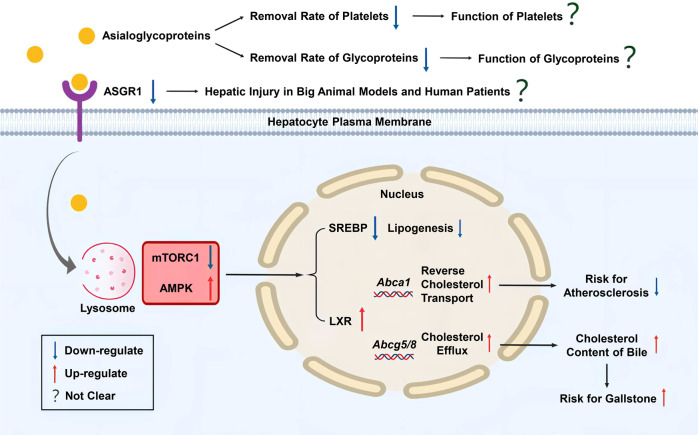
Mechanism and possible effects of downregulation of ASGR1. Downregulation of ASGR1 results in the AMPK activation and the mTORC1 inhibition, which leads to the downregulation of SREBP and the upregulation of LXR and triggers a series of reactions in cholesterol metabolism. Besides, the removal rate of asialoglycoproteins is reduced, which may affect the clearance of old glycoproteins and platelets. Figure was created with Biorender.com (http://biorender.com)

However, it is worth noting that the inhibition of ASGR1 to increase cholesterol efflux may also produce potential risks. By raising the cholesterol content of the bile, the chance of developing gallstones is likely to increase. Although the inhibition of ASGR1 in this study did not cause detectable liver injury in mice, the increase in cholesterol efflux changed the energy metabolism of hepatocytes. Therefore, future studies are warranted to elucidate whether ASGR1 inhibition causes liver injury in humans. Although hepatic injury has not been found in humans with ASGR1 variants, Xie et al.^4^ observed different degrees of hepatic injury in *Asgr1*-deficient pigs. Therefore, it is important to explore whether or not ASGR1 inhibition causes liver damage or other adverse effects in large animal models such as rhesus monkeys. In addition, ASGR1 reduction can inhibit the removal of old platelets and generation of new platelets. Therefore, inhibition of ASGR1 may affect the physiological function of platelets. Furthermore, because glycoproteins have a variety of roles in the body, the effect of inhibiting ASGR1 on the physiological function of its ligands must also be considered. Many glycoproteins have sialic acids residues as part of their structures, and their dessialylation is recognized by ASGR1 and dessialylation process causes them to be cleared by the liver. Therefore, ASGR1 is the key to maintain the delicate balance between the production and degradation of glycoproteins. In addition, since GalNAc is one of best described specific ligands of ASGR1, GalNAc-ASO therapy is currently being used in targeted drug delivery in the liver, exemplified by GalNAc-PCSK9-ASO and GalNAc-ANGPTL3-ASO. Therefore, the combination of ASGR1 inhibition with GalNAc-PCSK9-ASO or GalNAc-ANGPTL3-ASO should be discouraged. It remains to be elucidated in the future whether ASGR1 inhibition causes other potential risks (Fig. 1).

Looking forward, the study by Wang et al.^1^ ushers in a new research area of cholesterol-lowering therapies by targeting ASGR1 in hypercholesterolemia, cardiovascular and metabolic diseases. The study also provides tremendous impetus to develop small-molecule inhibitors of ASGR1 for clinical therapeutics.
